# Gaussian Process Based Expected Information Gain Computation for Bayesian Optimal Design

**DOI:** 10.3390/e22020258

**Published:** 2020-02-24

**Authors:** Zhihang Xu, Qifeng Liao

**Affiliations:** 1 School of Information Science and Technology, ShanghaiTech University, Shanghai 201210, China; xuzhh@shanghaitech.edu.cn; 2 Shanghai Institute of Microsystem and Information Technology, Chinese Academy of Sciences, Shanghai 200050, China; 3 University of Chinese Academy of Sciences, Beijing 100049, China

**Keywords:** Bayesian Monte Carlo, Bayesian optimal experimental design, Bayesian optimization

## Abstract

Optimal experimental design (OED) is of great significance in efficient Bayesian inversion. A popular choice of OED methods is based on maximizing the expected information gain (EIG), where expensive likelihood functions are typically involved. To reduce the computational cost, in this work, a novel double-loop Bayesian Monte Carlo (DLBMC) method is developed to efficiently compute the EIG, and a Bayesian optimization (BO) strategy is proposed to obtain its maximizer only using a small number of samples. For Bayesian Monte Carlo posed on uniform and normal distributions, our analysis provides explicit expressions for the mean estimates and the bounds of their variances. The accuracy and the efficiency of our DLBMC and BO based optimal design are validated and demonstrated with numerical experiments.

## 1. Introduction

As acquiring data in experiments is generally computationally demanding and time-consuming, maximizing the informativeness of experimental data is of crucial importance. For example, in the area of climate science, a complex system with various stochastic inputs is integrated to represent the real climate situation. Usually, the locations and the time of putting sensors to collect climate observations are optional. With limited resources, careful selections of sensor placements are required (see Reference [1] for a detailed discussion). Many works focus on finding experimental data carrying more information, and this topic is usually referred to as optimal experimental design (OED) [2]. In this paper, we consider the OED problem in the context of the Bayesian inverse problem. Given a forward problem, the inverse problem is to infer parameters inherent of the forward model through a set of design points and the corresponding responses. In the Bayesian setting, the parameters of interest are viewed as random variables, and hence the posterior distribution of the parameters can be obtained via the Bayes’ rules [3,4,5,6]. In linear cases, the OED problem includes various criteria such as the D-optimal design criterion and the A-optimal design criterion. The D-optimal design criterion seeks to maximize the determinant of the information matrix of the design, whereas the A-optimal design criterion considers minimizing the trace of the inverse of the information matrix which results in minimizing the average variance [7,8,9].

We focus on the decision theoretic approach that considers maximizing the expectation of Kullback-Leibler (KL) divergence from the posterior distribution to the prior distribution [10]. This decision theoretic approach is a nonlinear generalization of the Bayesian D-optimal criterion [11]. The objective function we want to maximize, the expectation of KL divergence, is also referred to as the expected information gain (EIG) over parameters. Computing EIG is usually a challenging problem, since it does not have an analytical form for nonlinear problems in general. In the literature, the following attempts have been made for this problem. Efficient surrogates for the forward models are introduced to make the problem tractable [1,12,13,14,15]. Recently, as the rise of novel computational methods, new approaches are actively developed to evaluate the EIG, including the quasi-Monte Carlo method [16] and the layered importance sampling [17] in the context of the focused optimal design. As the goal of this kind of optimal design is to find the maximizer of EIG, it is crucial to apply efficient optimization strategies. In Reference [12], a curve fitting surrogate of Monte Carlo experiments is proposed to result in an efficient optimization scheme. For the Bayesian optimal design problem focusing on the risk from an optimal terminal decision, the Bayesian optimization (BO) approach is proposed to find the minimizer of the risk [9]. We note that, while BO is proposed for the Bayesian optimal design problem for minimizing the risk in Reference [9], BO is considered for maximizing the EIG in this work. In addition, efficient approximate coordinate exchange strategies are proposed for Bayesian design in Reference [18]. For intractable likelihood models, Gaussian process (GP) models are built to emulate the likelihood function in Reference [19]. Gradient-based optimization methods are proposed to compute the maximizer of EIG in References [13,14]. Review of modern computational methods for decision theoretic optimal experimental design is provided in Reference [20].

The main purpose of this work is to propose an efficient Gaussian process (GP) based Bayesian optimal design strategy, where Bayesian Monte Carlo (BMC) and Bayesian optimization (BO) which are both based on GP are used [21,22,23]. As the Monte Carlo simulation for EIG involves an inner layer simulation and an outer layer simulation (see Reference [14]), we develop a novel efficient double-loop Bayesian Monte Carlo (DLBMC) method, which employs BMC [24,25,26] for both layers. However, the EIG is generally computationally expensive and its gradient information is typically not given explicitly. We propose a BO method [27,28,29] to compute the maximizer of EIG, where the gradient information of EIG is not required. To summarize, the contributions of this work are three-fold: first we develop a novel DLBMC to efficiently compute EIG; second we analyze the BMC for the normal and the uniform distributions; third we propose BO to obtain the maximizer of EIG.

This paper is organized as follows. In Section 2, we review the Bayesian optimal experimental design problem and formulate the expected information gain (EIG) criterion. In Section 3, we derive a double-loop Bayesian Monte Carlo estimator for the EIG and propose a Bayesian optimization approach to obtain the maximizer of the approximated EIG. Detailed analysis of BMC for the normal and the uniform distributions are conducted in this section. In Section 4, we demonstrate the efficiency of our GP based Bayesian optimal design with three test problems. Section 5 concludes the paper and provides discussions of the advantages and disadvantages of our algorithm.

## 2. Formulation of Experimental Design

In this section, we review the setting of the Bayesian optimal design problem following the presentation in [14]. In the Bayesian setting, the unknown parameters are viewed as random variables. Let (Ω,F,P) be a probability space, where Ω is a sample space, F is a σ-field, and P is the probability measure on (Ω,F). Let $\theta :\Omega \to R^{n_{\theta }}$ denote the parameters of interest, where $n_{\theta }$ is the dimension of the unknown parameters. Assume that θ is associated with a prior measure μ on $R^{n_{\theta }}$ satisfying $\mu \left(A\right)=P(\theta^{-1}\left(A\right))$ for $A\in R^{n_{\theta }}$. Throughout this paper, we assume that all the random variables have densities with respect to the Lebesgue measure. Let $d\in D\subset R^{n_{d}}$ denotes the design variable, where $n_{d}$ is the number of design variables and D denotes the design space. Let $y\in R^{n_{y}}$ denote the response associated with d where $n_{y}$ is the dimension of response. The inference of θ can be obtained based on the prior distribution and observations via Bayes’ rule,

(1) $$\underset{\mathrm{Posterior}}{\underset{︸}{p(\theta |d,y)}}=\frac{\underset{\overset{︷}{p(y|\theta ,d)}}{\mathrm{Likelihood}}\underset{\overset{︷}{p(\theta |d)}}{\mathrm{Prior}}}{\underset{\mathrm{Evidence}}{\underset{︸}{p(y|d)}}}\;.$$

The likelihood function is often determined by a deterministic forward model and a statistical model for measurement of model noises. Here we model the relation of the design variable and the observation by a deterministic model G(θ,d) and additive Gaussian noises ϵ,

(2) $$y=G\left(\theta ,d\right)+\epsilon \;,\;\epsilon ∼N\left(0,\sigma^{2}I\right)\;,$$

where G is the forward model. In many practical problems, the forward model is computationally expensive, and its explicit form is not given. So we can just view it as a black box whose internal structure is unknown, whereas we can generate noisy responses given fixed design variables and parameters.

Following the decision theoretic approach [10], we set the utility function as the KL divergence from the posterior distribution to the prior distribution,

(3) $$u\left(d,y,\theta \right)=D_{\mathrm{KL}}\left(p(\theta |d,y∥p\left(\theta \right))\right)=u\left(d,y\right)\;.$$

This term is actually independent of θ. Noting that u(d,y) is a function of both d and y, therefore we further take expectation of *u* over y to define the expected information gain (EIG):

$$\begin{array}{c} U\left(d\right)=\int_{Y}u\left(d,y\right)p\left(y|d\right)dy=\int_{Y}\int_{\Theta }p\left(\theta |d,y\right)\log\left[\frac{p(\theta |d,y)}{p(\theta )}\right]d\theta p\left(y|d\right)dy\;. \end{array}$$

Then the optimal experimental design is to find a design point which maximizes the expected utility, that is,

(4) $$d^{⋆}=\arg\max_{d\in D}U\left(d\right)\;.$$

A double-loop Monte Carlo (DLMC) estimator of EIG is proposed in [30]. Rewrite U(d) as

$$\begin{array}{cc} U(d)\; & =\int_{Y}\int_{\Theta }p\left(\theta |d,y\right)\log\left[\frac{p(\theta |d,y)}{p(\theta )}\right]d\theta p\left(y|d\right)dy\\ \; & =\int_{Y}\int_{\Theta }\left\{\log\left[p\left(y|\theta ,d\right)\right]-\log\left[p\left(y|d\right)\right]\right\}p\left(y,\theta |d\right)d\theta dy\;, \end{array}$$

and note that p(θ|d)=p(θ), since the specification of d does not provide further information about inference of θ. Then, the DLMC method approximates U(d) as

(5) $$U\left(d\right)\approx \frac{1}{n_{\mathrm{out}}}\sum_{i=1}^{n_{\mathrm{out}}}\left[\log\left(p\left(y^{\left(i\right)}|\theta^{\left(i\right)},d\right)\right)-\log\left(p\left(y^{\left(i\right)}|d\right)\right)\right]\;,$$

where $\theta^{\left(i\right)}$ are drawn from the prior p(θ), and $y^{\left(i\right)}$ are drawn from the conditional distribution $p(y|\theta =\theta^{\left(i\right)},d)$ (i.e., the likelihood), and hence $p(y^{\left(i\right)}|d)$ can be estimated via the importance sampling technique,

(6) $$p\left(y^{\left(i\right)}|d\right)=\int_{\Theta }p\left(y^{\left(i\right)}|\theta ,d\right)p\left(\theta \right)d\theta \approx \frac{1}{n_{\mathrm{in}}}\sum_{j=1}^{n_{\mathrm{in}}}p\left(y^{\left(i\right)}|\theta^{\left(i,j\right)},d\right)\;.$$

Combining (6) and (5) yields a biased estimator $\tilde{U}\left(d\right)$ of U(d). However, if we sample ${\left\{\theta^{\left(i,j\right)}\right\}}_{j=1}^{n_{\mathrm{in}}}$ for every $y^{\left(i\right)}$ ($i=1,\ldots ,n_{\mathrm{out}}$), the complexity of this method is $O(n_{\mathrm{out}}n_{\mathrm{in}})$. To reduce the computational cost, a sample reuse technique is employed in [14]. That is, for every d, we drawn a fresh batch from the prior ${\left\{\theta^{\left(k\right)}\right\}}_{k=1}^{n_{\mathrm{out}}}$ and use this set for both the outer Monte Carlo and the inner Monte Carlo (i.e., $\theta^{\left(\cdot ,j\right)}=\theta^{\left(k\right)}$). Consequently, the complexity is reduced to $O(n_{\mathrm{out}})$.

## 3. GP Based Framework for Bayesian Optimal Design

Our main framework for Bayesian optimal design is based on two powerful tools according to Gaussian processes: the Bayesian Monte Carlo (BMC) method and the Bayesian optimization (BO) method. In this section, we first review BMC and conduct the analysis of BMC for the normal and the uniform distributions, and then present our novel double loop Bayesian Monte Carlo (DLBMC) for EIG. After that, we propose BO to find the maximizer of the approximated EIG. Finally, we review the classical Markov chain Monte Carlo (MCMC) method for Bayesian parameter inference.

### 3.1. Bayesian Monte Carlo

Consider the integral problem $I:=\int_{X}f\left(x\right)p\left(x\right)dx\;,$ where p(x) is the density of *x*, $f\left(x\right):R^{n}\to R$ is the integrand, and X is the support of *x*, that is, the integration domain. The idea of BMC is to formulate an integral problem into a Bayesian inference problem by placing a prior over the integrand *f* and to obtain the posterior distribution of *f* conditioning on the data collected. A natural way of putting a prior over function is through a Gaussian process, which is completely characterized by its mean function μ(x) and kernel function $k(x,x^{\prime })$, that is, f∼GP(μ(·),k(·,·)). A commonly used choice for the kernel function is the Gaussian kernel (or known as squared exponential kernel), $k\left(x,x^{\prime }\right)=\sigma_{f}^{2}\exp(-∥x-x^{\prime }{∥}_{2}^{2}/\left(2l^{2}\right))$, where both $\sigma_{f}$ and *l* are hyperparameters of the kernel function. The choice of hyperparameters affects the result to a large extent. Therefore the hyperparameters need to be determined carefully. Having collected noisy observations $D={\left\{x^{\left(i\right)},f^{\left(i\right)}\right\}}_{i=1}^{N}$, where $f^{\left(i\right)}=f\left(x^{\left(i\right)}\right)+\epsilon^{\left(i\right)}$ with Gaussian noises $\epsilon^{\left(i\right)}∼N\left(0,\sigma^{2}\right)\;,$ a Gaussian process provides a posterior distribution for an arbitrary new point $x^{⋆}$, $f\left(x^{⋆}\right)|D,x^{⋆}∼N\left(\mu_{N}\left(x^{⋆}\right),\sigma_{N}\left(x^{⋆}\right)\right)\;,$ with mean function $\mu_{N}\left(x^{⋆}\right)$ and variance function $\sigma_{N}\left(x^{⋆}\right)$ given by

$$\begin{array}{cc} \; & \mu_{N}\left(x^{⋆}\right):=k_{N}{\left(x^{⋆}\right)}^{T}{\left(K_{N}+\sigma^{2}I\right)}^{-1}f_{N}\;,\\ \; & \sigma_{N}\left(x^{⋆}\right):=k\left(x^{⋆},x^{⋆}\right)-k_{N}{\left(x^{⋆}\right)}^{T}{\left(K_{N}+\sigma^{2}I\right)}^{-1}k_{N}\left(x^{⋆}\right)\;, \end{array}$$

where $k_{N}\left(x^{⋆}\right)={\left[k\left(x^{⋆},x^{\left(i\right)}\right)\right]}_{N\times 1}$, $K_{N}={\left[k\left(x^{\left(i\right)},x^{\left(j\right)}\right)\right]}_{N\times N}$, and $f_{N}={\left[f^{\left(i\right)}\right]}_{N\times 1}\;.$ In some special cases (For example, when *x* is distributed with Gaussian and the kernel is a Gaussian kernel [25]), GP allows us to estimate the integration in a closed form, of which the posterior mean is given by,

(7) $$\begin{array}{cc} E_{f|D}\left[I\right]\; & ={\;\int \int \;}_{X}f\left(x\right)p\left(x\right)dxp\left(f|D\right)df=\int_{X}\left[\int f(x)p(f|D)df\right]p\left(x\right)dx\\ \; & =\int_{X}E_{f|D}\left(f\right)p\left(x\right)dx=\int_{X}\mu_{N}\left(x\right)p\left(x\right)dx=\int_{X}k_{N}{\left(x\right)}^{T}{\left(K_{N}+\sigma^{2}I\right)}^{-1}f_{N}p\left(x\right)dx\\ \; & =\int_{X}k_{N}{\left(x\right)}^{T}p\left(x\right)dx{\left(K_{N}+\sigma^{2}I\right)}^{-1}f_{N}=z^{T}{\left(K_{N}+\sigma^{2}I\right)}^{-1}f_{N}\;, \end{array}$$

where $z:=\int_{X}k_{N}{\left(x\right)}^{T}p\left(x\right)dx$, and the posterior variance is given by

(8) $$\begin{array}{cc} \; & V_{f|D}\left[I\right]=E_{f|D}\left\{{\left[I-E_{f|D}\left(I\right)\right]}^{2}\right\}=\int {\left[\int_{X}f\left(x\right)p\left(x\right)dx-\int_{X}\mu_{N}\left(x^{\prime }\right)p\left(x^{\prime }\right)dx^{\prime }\right]}^{2}p\left(f|D\right)df\\ \; & \;={\;\int \int \;}_{X\times X}\int {\left[f\left(x\right)-\mu_{N}\left(x^{\prime }\right)\right]}^{2}p\left(f|D\right)dfp\left(x\right)p\left(x^{\prime }\right)dxdx^{\prime }={\;\int \int \;}_{X\times X}V\left(f\left(x\right)\right)p\left(x\right)p\left(x^{\prime }\right)dxdx^{\prime }\\ \; & \;={\;\int \int \;}_{X\times X}k\left(x,x^{\prime }\right)p\left(x\right)p\left(x^{\prime }\right)dxdx^{\prime }-z^{T}{\left(K_{N}+\sigma^{2}I\right)}^{-1}z\;. \end{array}$$

Note that in the above analytical expression of posterior mean and variance, when the hyperparameters and the kernel function are given, *z* and *K* are determined by ${\left\{x^{\left(i\right)}\right\}}_{i=1}^{N}$ and they are independent of the observations ${\left\{f^{\left(i\right)}\right\}}_{i=1}^{N}$. Therefore, the computation procedure for the posterior mean and variance of the BMC estimator proceeds the following two steps: first, an input sample set ${\left\{x^{\left(i\right)}\right\}}_{i=1}^{N}$ is generated, and *z* and *K* are computed; second, the corresponding observation set ${\left\{f^{\left(i\right)}\right\}}_{i=1}^{N}$ is collected, and the posterior mean and variance are computed through (7) and (8) respectively. Next, when *x* is an uniform or Gaussian random vector, we provide detailed derivations for the posterior mean and variance of BMC estimators.

**Theorem** **1** (Bayesian Monte Carlo for the standard Gaussian distribution). *Consider the integral $I\;=\;\int_{X}f\left(x\right)p\left(x\right)dx$, where x is the standard Gaussian random variable vector in $X=R^{n}$. The prior mean function is assumed to be a zero function, and the kernel is assumed to be the Gaussian kernel $k\left(x,x^{\prime }\right)\;=\sigma_{f}^{2}\exp(-∥x-x^{\prime }{∥}_{2}^{2}/\left(2l^{2}\right))$ with predetermined hyperparameters $\sigma_{f}$ and l. Having collected noisy observations $D={\left\{x^{\left(i\right)},f^{\left(i\right)}\right\}}_{i=1}^{N}$ where $f^{\left(i\right)}=f\left(x^{\left(i\right)}\right)+\epsilon^{\left(i\right)}$ and $\epsilon^{\left(i\right)}∼N\left(0,\sigma^{2}\right)$, the posterior mean and variance of BMC are given by*

$$\begin{array}{cc} \; & E_{f|D}\left[I\right]=z^{T}{\left(K_{N}+\sigma^{2}I\right)}^{-1}f_{N}\;,\\ \; & V_{f|D}\left[I\right]=\sigma_{f}^{2}{\left(\frac{l^{2}}{l^{2}+2}\right)}^{n/2}-z^{T}{\left(K_{N}+\sigma^{2}I\right)}^{-1}z\;, \end{array}$$

*where $K_{N}={\left[k\left(x^{\left(i\right)},x^{\left(j\right)}\right)\right]}_{N\times N}$, $f_{N}={\left[f^{\left(1\right)},\ldots ,f^{\left(N\right)}\right]}_{N\times 1}$, and the components of z are*

(9) $$z_{i}=\sigma_{f}^{2}{\left(\frac{l^{2}}{l^{2}+1}\right)}^{n/2}\exp\left(-\frac{∥x^{\left(i\right)}{∥}^{2}}{2(l^{2}+1)}\right)\;,$$

*for i=1,…,N.*

**Proof.**  The components of *z* can be computed analytically, for i=1,…,N,

$$\begin{array}{cc} z_{i}\; & =\int_{X}\sigma_{f}^{2}\exp\left(-\frac{∥x-x^{\left(i\right)}{∥}_{2}^{2}}{2l^{2}}\right)\frac{1}{\sqrt{{\left(2\pi \right)}^{n}}}\exp\left(-\frac{x^{T}x}{2}\right)dx\\ \; & =\frac{\sigma_{f}^{2}}{\sqrt{{\left(2\pi \right)}^{n}}}\int_{X}\exp\left[-\frac{1}{2}x^{T}\frac{(l^{2}+1)I}{l^{2}}x+\frac{x^{T}x^{\left(i\right)}}{l^{2}}-\frac{∥x^{\left(i\right)}{∥}^{2}}{2l^{2}}\right]dx\\ \; & =\sigma_{f}^{2}{\left|\frac{l^{2}}{l^{2}+1}I\right|}^{1/2}\exp\left[-\frac{∥x^{\left(i\right)}{∥}^{2}}{2l^{2}}-\frac{∥x^{\left(i\right)}{∥}_{2}^{2}}{2l^{2}\left(l^{2}+1\right)}\right]\\ \; & =\sigma_{f}^{2}{\left(\frac{l^{2}}{l^{2}+1}\right)}^{n/2}\exp\left(-\frac{∥x^{\left(i\right)}{∥}_{2}^{2}}{2(l^{2}+1)}\right)\;. \end{array}$$

Moreover, the variance of BMC is

$$\begin{array}{cc} V_{f|D}\left[I\right]\; & ={\;\int \int \;}_{X\times X}k\left(x,x^{\prime }\right)p\left(x\right)dxp\left(x^{\prime }\right)dx^{\prime }-z^{T}K_{N}^{-1}z\\ \; & =\int_{X}\frac{\sigma_{f}^{2}}{{\left(2\pi \right)}^{n/2}}{\left(2\pi \right)}^{n/2}{\left|\frac{l^{2}}{l^{2}+1}I\right|}^{1/2}\exp\left(-\frac{∥x^{\prime }{∥}_{2}^{2}}{2l^{2}}+\frac{∥x^{\prime }{∥}_{2}^{2}}{2l^{2}\left(l^{2}+1\right)}\right)p\left(x^{\prime }\right)dx-z^{T}K_{N}^{-1}z\\ \; & =\sigma_{f}^{2}{\left(\frac{l^{2}}{l^{2}+1}\right)}^{n/2}\int_{X}\frac{1}{{\left(2\pi \right)}^{n/2}}\exp\left(-\frac{l^{2}+2}{2(l^{2}+1)}{∥x^{\prime }∥}_{2}^{2}\right)dx-z^{T}K_{N}^{-1}z\\ \; & =\sigma_{f}^{2}{\left(\frac{l^{2}}{l^{2}+1}\right)}^{n/2}\frac{1}{{\left(2\pi \right)}^{n/2}}{\left(2\pi \right)}^{n/2}{\left(\frac{l^{2}+1}{l^{2}+2}\right)}^{n/2}-z^{T}K_{N}^{-1}z\\ \; & =\sigma_{f}^{2}{\left(\frac{l^{2}}{l^{2}+2}\right)}^{n/2}-z^{T}K_{N}^{-1}z\;. \end{array}$$

 □

We note that Theorem 1 is presented in References [22,25], but we give the above detailed proof for completeness.

**Theorem** **2** (Bayesian Monte Carlo for the uniform distribution). *Consider the integral $I=\int_{X}f\left(x\right)p\left(x\right)dx$, where x is a random vector uniformly distributed in the hypercube $X=\left[l_{1},r_{1}\right]\times \cdots \times \left[l_{n},r_{n}\right]$. The prior mean function is assumed to be a zero function, and the kernel is assumed to be the Gaussian kernel $k\left(x,x^{\prime }\right)=\sigma_{f}^{2}\exp(-∥x-x^{\prime }{∥}_{2}^{2}/\left(2l^{2}\right))$ with predetermined hyperparameters $\sigma_{f}$ and l. Having collected noisy observations $D={\left\{x^{\left(i\right)},f^{\left(i\right)}\right\}}_{i=1}^{N}$ where $f^{\left(i\right)}=f\left(x^{\left(i\right)}\right)+\epsilon^{\left(i\right)}$ and $\epsilon^{\left(i\right)}∼N\left(0,\sigma^{2}\right)$, the posterior mean of BMC and the upper bound of variance of BMC are given by*

$$\begin{array}{cc} \; & E_{f|D}\left[I\right]=z^{T}\left(K_{N}+\sigma^{2}I\right)f\;,\\ \; & V_{f|D}\left[I\right]<\frac{\sigma_{f}^{2}\sqrt{{\left(2\pi l^{2}\right)}^{n}}}{\left|X\right|}-z^{T}\left(K_{N}+\sigma^{2}I\right)z\;, \end{array}$$

*where $K_{N}={\left[k\left(x^{\left(i\right)},x^{\left(j\right)}\right)\right]}_{N\times N}$, $f_{N}={\left[f^{\left(1\right)},\ldots ,f^{\left(N\right)}\right]}_{N\times 1}$, and the components of z are given by*

(10) $$z_{i}=\frac{\sigma_{f}^{2}}{\left|X\right|}\prod_{j=1}^{n}\left(\Phi \left(x_{j};x_{j}^{\left(i\right)},l,r_{j}\right)-\Phi \left(x_{j};x_{j}^{\left(i\right)},l,l_{j}\right)\right)\;,$$

*for i=1,…,N, with Φ(x;μ,σ,t) being the cumulative distribution function (CDF) of the Gaussian distribution $N(\mu ,\sigma^{2})$.*

**Proof.**  The components of *z* can be computed analytically, for i=1,…,N,

$$\begin{array}{cc} z_{i} & =\int_{X}k\left(x,x^{\left(i\right)}\right)p\left(x\right)dx=\frac{\sigma_{f}^{2}}{\left|X\right|}\int_{X}\exp\left(-\frac{∥x-x^{\left(i\right)}{|∥}^{2}}{2l^{2}}\right)dx\\ \; & =\frac{\sigma_{f}^{2}}{\left|X\right|}\int_{X}\exp\left(-\frac{\sum_{j=1}^{n}{\left(x_{j}-x_{j}^{\left(i\right)}\right)}^{2}}{2l^{2}}\right)dx=\frac{\sigma_{f}^{2}}{\left|X\right|}\prod_{j=1}^{n}\int_{l_{j}}^{r_{j}}\exp\left(-\frac{{\left(x_{j}-x_{j}^{\left(i\right)}\right)}^{2}}{2l^{2}}\right)dx_{j}\\ \; & =\frac{\sigma_{f}^{2}}{\left|X\right|}\prod_{j=1}^{n}\left(\Phi \left(x_{j};x_{j}^{\left(i\right)},l,r_{j}\right)-\Phi \left(x_{j};x_{j}^{\left(i\right)},l,l_{j}\right)\right)\;, \end{array}$$

where $\Phi \left(x;\mu ,\sigma ,t\right)=\int_{\infty }^{t}\frac{1}{\sigma \sqrt{2\pi }}\exp\left(-\frac{{\left(x-\mu \right)}^{2}}{2\sigma^{2}}\right)dx$ is the CDF of the Gaussian distribution $N(\mu ,\sigma^{2})$ and $v_{j}$ denotes the *j*-th component of the vector *v*. However, since the double integral of the Gaussian density function has no analytical form, the variance of the estimator cannot be obtained, and we give an upper bound of the variance,

$$\begin{array}{cc} V_{f|D}\left[I\right]\; & =\int_{X}\int_{X}k\left(x,x^{\prime }\right)p\left(x\right)dxp\left(x^{\prime }\right)dx^{\prime }-z^{T}K_{N}^{-1}z<\int_{X}\int_{R^{n}}k\left(x,x^{\prime }\right)p\left(x\right)dxp\left(x^{\prime }\right)dx^{\prime }-z^{T}K_{N}^{-1}z\\ \; & =\frac{\sigma_{f}^{2}\sqrt{{\left(2\pi l^{2}\right)}^{n}}}{\left|X\right|}\int_{X}p\left(x^{\prime }\right)dx^{\prime }-z^{T}K_{N}^{-1}z=\frac{\sigma_{f}^{2}\sqrt{{\left(2\pi l^{2}\right)}^{n}}}{\left|X\right|}-z^{T}K_{N}^{-1}z\;. \end{array}$$

 □

### 3.2. Estimating the Expected Information Gain Using Double-Loop BMC

In general, the value of the EIG has no closed form and has to be approximated via numerical methods. Based on the idea of BMC for efficiently evaluating integrals, we develop a double-loop BMC (DLBMC) scheme to approximate the EIG.

Letting e(y,d)=p(y|d), g(d,y,θ)={ln[p(y|θ,d)]−ln[p(y|d)]}p(y|θ,d)={ln[p(y|θ,d)]−ln[e(y,d)]}p(y|θ,d), and $h\left(d,y\right)=\int_{\Theta }g\left(d,y,\theta \right)p\left(\theta \right)d\theta$, it is known that U(d) can be rewritten as

$$\begin{array}{cc} U(d)\; & =\int_{Y}\int_{\Theta }\left\{\ln\left[p\left(y|\theta ,d\right)\right]-\ln\left[e\left(y,d\right)\right]\right\}p\left(y|\theta ,d\right)p\left(\theta \right)d\theta dy\\ \; & =\int_{Y}\int_{\Theta }g\left(d,y,\theta \right)p\left(\theta \right)d\theta dy=\int_{Y}h\left(d,y\right)dy\;. \end{array}$$

First, we consider the straightforward detailed calculation of U(d) for any fixed d. To compute $U\left(d\right)=\int_{Y}h\left(d,y\right)dy$, we need samples ${\left\{y^{\left(i\right)},h^{\left(i\right)}:=h\left(d,y^{\left(i\right)}\right)\right\}}_{i=1}^{n_{\mathrm{out}}}$, where $y^{\left(i\right)}∼U\left(Y\right)$ for $i\;=\;1,\ldots ,n_{\mathrm{out}}$, $n_{\mathrm{out}}$ denotes the sample size of the outer layer. To compute $h^{\left(i\right)}:=\int_{\Theta }g\left(d,y^{\left(i\right)},\theta \right)p\left(\theta \right)d\theta$, samples ${\left\{\theta^{\left(i,j\right)},g^{\left(i,j\right)}:=g\left(d,y^{\left(i\right)},\theta^{\left(i,j\right)}\right)\right\}}_{j=1}^{n_{\mathrm{in}}}$ are needed, where $n_{\mathrm{in}}$ denotes the sample size of the inner layer. Again, $g^{\left(i,j\right)}=\left\{\ln\left[p\left(y^{\left(i\right)}|\theta^{\left(i,j\right)},d\right)\right]-\ln\left[p\left(y^{\left(i\right)}|d\right)\right]\right\}p\left(y^{\left(i\right)}|\theta^{\left(i,j\right)},d\right)$ also involves another integration $p\left(y^{\left(i\right)}|d\right)=\int_{\Theta }p\left(y^{\left(i\right)}|d,\theta \right)p\left(\theta \right)d\theta$, and therefore samples ${\left\{\theta^{\left(i,j\right)},p\left(y^{\left(i\right)}|d,\theta^{\left(i,j\right)}\right)\right\}}_{j=1}^{n_{\mathrm{in}}}$ are needed and $\theta^{\left(i,j\right)}$ are generated from the prior. We propose using the BMC method to evaluate integrals ${\left\{e^{\left(i\right)}\right\}}_{i=1}^{n_{\mathrm{out}}}$, ${\left\{h^{\left(i\right)}\right\}}_{i=1}^{n_{\mathrm{out}}}$, and *U*. So far, there are two problems. First, the computation complexity for the forward model is $O(n_{\mathrm{in}}n_{\mathrm{out}})$, which grows fast with the increase of the problem dimension. Second, since we usually have no prior knowledge of the support Y of y, we can not uniformly sample ${\left\{y^{\left(i\right)}\right\}}_{i=1}^{n_{\mathrm{out}}}$.

To overcome these obstacles, we employ the sample reuse technique [14] that sets $\theta^{\left(\cdot ,j\right)}=\theta^{\left(j\right)}$, and the computational complexity is reduced to $O(n_{\mathrm{in}})$. Besides, it allows us to generate samples ${\left\{\theta^{\left(j\right)}\right\}}_{j=1}^{n_{\mathrm{in}}}$ in advance, and we can use the corresponding forward model outputs to estimate Y—suppose the corresponding forward model values are given by ${\left\{G^{\left(j\right)}=G\left(d,\theta^{\left(j\right)}\right)\right\}}_{j=1}^{n_{\mathrm{in}}}$, and then Y can be approximated by $\tilde{Y}:=\left[\min\left(G\right)-\sigma ,\max\left(G\right)+\sigma \right]$ where $G={\left[G^{\left(1\right)},\ldots ,G^{n_{\mathrm{in}}}\right]}^{T}$. In this way, we can sample ${\left\{y^{\left(i\right)}\right\}}_{i=1}^{n_{\mathrm{in}}}∼U\left(\tilde{Y}\right)$. Intuitively speaking, the approximated $\tilde{Y}$ is slightly smaller than the actual field Y, and consequently, bias is induced in the estimator. With increased sample size $n_{\mathrm{in}}$, $\tilde{Y}$ can be captured more accurately and the bias can be reduced.

In the process of estimating *U*, we propose using the BMC method to compute *e*, *h* and *U*. Since two layers of integration are involved, let the hyperparameters of BMC for the inner layer and the outer layer be $\left\{l_{\mathrm{in}},{\left(\sigma_{f}\right)}_{\mathrm{in}}\right\}$ and $\left\{l_{\mathrm{out}},{\left(\sigma_{f}\right)}_{\mathrm{out}}\right\}$ respectively. In our previous discussion about BMC in (7)–(8), *z* and *K* can be computed, once the input sample set ${\left\{x^{\left(i\right)}\right\}}_{i=1}^{N}$ is given. Therefore, for computational simplicity, after ${\left\{\theta^{\left(j\right)}\right\}}_{j=1}^{n_{\mathrm{in}}}$ and ${\left\{y^{\left(i\right)}\right\}}_{i=1}^{n_{\mathrm{out}}}$ are generated, we can compute $\left\{z_{\mathrm{in}},K_{\mathrm{in}}\right\}$ and $\left\{z_{\mathrm{out}},K_{\mathrm{out}}\right\}$ ahead. Taking the prior being the standard normal distribution for example, $z_{\mathrm{in}}$ and $K_{\mathrm{in}}$ are given by,

$$\begin{array}{cc} \; & {\left(z_{\mathrm{in}}\right)}_{j}={\left(\sigma_{f}\right)}_{\mathrm{in}}^{2}{\left(\frac{l_{\mathrm{in}}^{2}}{l_{\mathrm{in}}^{2}+1}\right)}^{n_{\theta }/2}\exp\left(-\frac{∥\theta^{\left(j\right)}{∥}_{2}^{2}}{2(l_{\mathrm{in}}^{2}+1)}\right)\;,\;\mathrm{for}\;j=1,\ldots ,n_{\mathrm{in}}\;,\\ \; & K_{\mathrm{in}}=[{\left(\sigma_{f}\right)}_{\mathrm{in}}^{2}\exp(-∥\theta^{\left(i\right)}-\theta^{\left(j\right)}{∥}_{2}^{2}/\left(2l_{\mathrm{in}}^{2}\right){)]}_{n_{\mathrm{in}}\times n_{\mathrm{in}}}\;. \end{array}$$

Details of our DLBMC method for estimating *U* are summarized in Algorithm 1. Note that we only use the mean estimates of the DLBMC estimator in the following. The variance of DLBMC is potentially useful, but as discussed in Section 3.1, the variance of BMC typically does not have a closed form, and we are not able to derive a closed form for the variance of DLBMC in this work. We will consider the variance of DLBMC in our future work. It is also possible to consider other numerical integration methods to compute EIG, for example, the sparse grid quadrature rules [31,32] and their combination with physical model reduction techniques [33], but they are out of the scope of this paper.
**Algorithm 1** Double-Loop Bayesian Monte Carlo (DLBMC) for estimating EIG1:**Input:** Design points d, prior p(θ), standard deviation of noise σ, hyperparameters $\left\{l_{\mathrm{in}},{\left(\sigma_{f}\right)}_{\mathrm{in}}\right\}$ and $\left\{l_{\mathrm{out}},{\left(\sigma_{f}\right)}_{\mathrm{out}}\right\}$.2:**Data preparation:** Sample ${\left\{\theta^{\left(j\right)}\right\}}_{j=1}^{n_{\mathrm{in}}}∼p\left(\theta \right)$ and compute $G^{\left(j\right)}=G\left(d,\theta^{\left(j\right)}\right)$ for $j=1,\ldots ,n_{\mathrm{in}}$.3:Sample ${\left\{y^{\left(i\right)}\right\}}_{i=1}^{n_{\mathrm{out}}}∼U\left(\min\left(G\right)-\sigma ,\max\left(G\right)+\sigma \right)$.4:Compute $\left\{z_{\mathrm{in}},K_{\mathrm{in}}\right\}$ and $\left\{z_{\mathrm{out}},K_{\mathrm{out}}\right\}$.5:**for**$i=1,\ldots ,n_{\mathrm{out}}$**do**6:    **for** $j=1,\ldots ,n_{\mathrm{in}}$ **do**7:        Compute the likelihood $f^{\left(i,j\right)}=p\left(y^{\left(i\right)}|\theta^{\left(j\right)},d\right)$.8:    **end for**9:    Let $f^{\left(i\right)}={\left[f^{\left(i,1\right)},\ldots ,f^{\left(i,n_{\mathrm{in}}\right)}\right]}^{T}$.10:    Compute the evidence $e^{\left(i\right)}=z_{\mathrm{in}}^{T}K_{\mathrm{in}}^{-1}f^{\left(i\right)}$.11:    **for** $j=1,\ldots ,n_{\mathrm{in}}$ **do**12:        $g^{\left(i,j\right)}=\left[\log\left(f^{\left(i,j\right)}\right)-\log\left(e^{\left(i\right)}\right)\right]f^{\left(i,j\right)}\;.$13:    **end for**14:    Let $g^{\left(i\right)}={\left[g^{\left(i,1\right)},\ldots ,g^{\left(i,n_{\mathrm{in}}\right)}\right]}^{T}$.15:    Compute $h^{\left(i\right)}=z_{\mathrm{in}}^{T}K_{\mathrm{in}}g^{\left(i\right)}$.16:**end for**17:Let $h={\left[h^{\left(1\right)},\ldots ,h^{\left(n_{\mathrm{out}}\right)}\right]}^{T}$.18:Compute $\hat{U}\left(d\right)=z_{\mathrm{out}}^{T}K_{\mathrm{out}}^{-1}h\;.$19:**Output:** the estimated EIG $\hat{U}\left(d\right)$.

### 3.3. Bayesian Optimization

The ultimate goal of the optimal experimental design problem is to find the optimizer $d^{⋆}$ in (4). In this problem, since the computing of the function value U(d) and the gradient ∇U is prohibitively expensive, it is challenging to apply function-value-based or gradient-based optimization methods. As the Bayesian optimization (BO) method [27,28,34,35] typically only requires a low objective function evaluation budget [36] and does not require any gradient information, it can be suitable for this problem. In this section, we give a brief review of BO and apply it to obtain the maximizer of EIG (4).

To compute the maximizer of EIG $U:R^{n_{d}}\to R$ (see (4)), for a given maximum number of iterations $t_{\max}$, that is, the evaluation budget, Bayesian optimization begins with putting a GP prior on $U∼\mathrm{GP}(\mu_{0}\left(d\right),k\left(d,d^{\prime }\right))$, and then randomly chooses an initial point $d_{1}$ and collects the corresponding response $U_{1}=U\left(d_{1}\right)$. Next, the posterior mean function $\mu_{1}\left(d\right)$ and variance $\sigma_{1}\left(d\right)$ are updated via collected data set $S_{1}=\left\{d_{1},U_{1}\right\}$. Usually $d_{1}$ alone is inadequate to find the maximum and therefore we need a strategy to choose the next design point. Typically, the next point is determined through maximizing an acquisition function *A*, that is, at *t*-th iteration, $d_{t+1}=\arg\max_{d}A\left(d|S_{1:t}\right)$. After the next point $d_{2}$ is obtained, we sample the objective function $U_{2}$. The collected data set is then augmented as $S_{1:2}=S_{1}\cup \left\{d_{2},U_{2}\right\}={\left\{d_{i},U_{i}\right\}}_{i=1,2}$, and the posterior mean function $\mu_{2}\left(d\right)$ and the variance function $\sigma_{t}\left(d\right)$ are also updated. The above procedure repeats until the given maximum budget $t_{\max}$ is reached.

In the case that D is infinite, the process of finding the next design point $d_{t+1}=\arg\max_{d\in D}A\left(d|S_{1:t}\right)$ is demanding. However, performing global search over the discretized space is usually effective [27,37], since we assume that evaluating *U* is more costly than computing the GP surrogate. Therefore, the design space D is discretized over equidistant grids and we denote the discretized design space as $\bar{D}$. Supposing we have collected data set $S_{1:t}$, the posterior of *U* is a GP distribution with mean $\mu_{t}\left(d\right)$, kernel $k(d,d^{\prime })$ and variance $\sigma_{t}^{2}\left(d\right)$,

(11) $$\begin{array}{cc} \; & \mu_{t}\left(d\right)=k_{t}{\left(d\right)}^{T}{\left(K_{t}+\sigma^{2}I\right)}^{-1}U_{1:t}\;, \end{array}$$

(12) $$\begin{array}{cc} \; & \sigma_{t}\left(d\right)=k\left(d,d\right)-K_{t}{\left(d\right)}^{T}{\left(K_{t}+\sigma^{2}I\right)}^{-1}k_{t}\left(d\right)\;, \end{array}$$

where $k_{t}\left(d\right)={\left[k\left(d,d_{i}\right)\right]}_{t\times 1}$, $K_{t}={\left[k\left(d_{i},d_{j}\right)\right]}_{t\times t}$ and $U_{1:t}={\left[U_{i}\right]}_{t\times 1}$. We note the design space considered in this paper is assumed to be bounded, such that it can be directly discretized. For unbounded design spaces, an unbounded Bayesian optimization approach is developed through gradually extending regions with regularization in [38].

Choosing a proper acquisition function is crucial for the Bayesian optimization algorithm since it guides the search for the optimum. Popular choices of acquisition function include maximizing the probability of improvement (PI) [39,40], and maximizing the expected improvement (EI) in the efficient global optimization (EGO) algorithm [41,42]. A review for the selection of acquisition functions is in [27]. Suggested by [37], we apply the GP-UCB algorithm to choose the next point—the acquisition function is set to a linear combination of the posterior mean function and the posterior variance function,

$$d_{t}=\arg\max_{d\in D}\mu_{t-1}\left(d\right)+\sqrt{\beta_{t-1}}\sigma_{t-1}\left(d\right)\;,$$

where $\mu_{t-1}\left(d\right)+\sqrt{\beta_{t-1}}\sigma_{t-1}\left(d\right)$ can be considered as the upper confidence bound of the current Gaussian process. It is clear that maximizing the acquisition function $\mu_{t-1}\left(d\right)+\sqrt{\beta_{t-1}}\sigma_{t-1}\left(d\right)$ shows a trade-off between exploring the point with potential high function value and exploiting the point with high uncertainty. Here, $\beta_{t-1}$ is the parameter balancing exploring and exploiting. Details of our BO strategy for optimal design are shown in Algorithm 2.

We set the prior mean function to $\mu_{0}\left(d\right)=0$, and set the kernel to be the Gaussian kernel given by $k\left(d,d^{\prime }\right)=\sigma_{f}^{2}\exp(-∥d-d^{\prime }{∥}^{2}/\left(2l^{2}\right))\;.$ The design space D is discretized over equidistant grids and we denote the discretized design space as $\bar{D}$. In the step of maximizing the acquisition function, $d_{t}$ is located through a grid search over $\bar{D}$.

A natural measure in performance of the Bayesian optimization method is defined through average cumulative regret. Supposing the maximum $U(d^{⋆})$ is known, the instantaneous regret for iteration *t* is defined as $r_{t}=U\left(d^{⋆}\right)-U\left(d_{t}\right)$ and the cumulative regret $R_{T}$ after *T* iterations is defined as the sum of the instantaneous regrets $R_{T}=\sum_{t=1}^{T}r_{t}$. Then the average cumulative regret $R_{T}/T$ is defined as $R_{T}/T=\sum_{t=1}^{T}r_{t}/T$. It should be noted that neither $r_{t}$ nor $R_{T}$ can be obtained directly from the Algorithm 2. In [37], it is proven that, for finite design space $\bar{D}$, setting δ∈(0,1) and $\beta_{t}=2\log(|\bar{D}\left|t^{2}\pi^{2}/6\delta \right)$, the Bayesian optimization method is no-regret with high probability, that is, $\lim_{T\to \infty }R_{T}/T=0\;.$
**Algorithm 2** Bayesian optimization (BO) for optimal design1:**Input:** Design space D and its discretized design space $\bar{D}$, prior $\mu_{0}\left(d\right)=0$, hyperparameters $l,\sigma_{f}$ of the Gaussian kernel, hyperparameter δ, and maximum number of iterations $t_{\max}$.2:**for**$t=1,\ldots ,t_{\max}$**do**3:    Find the maximizer of the acquisition function: $d_{t}=\arg\max_{d\in D}\mu_{t-1}\left(d\right)+\sqrt{\beta_{t-1}}\sigma_{t-1}\left(d\right)$.4:    Sample the objective function $U_{t}=\hat{U}\left(d_{t}\right)$ using Algorithm 1.5:    Augment the data set $S_{1:t}={\left\{d_{i},U_{i}\right\}}_{i=1}^{t}$.6:    Perform Bayesian update to obtain $\mu_{t}$ and $\sigma_{t}$ over $\bar{D}$ using (11) and (12) respectively.7:    Update $\beta_{t}\;.$8:**end for**9:**Output:** Optimal design: $d^{⋆}=\arg\max_{t=1,\ldots ,t_{\max}}U_{t}$

### 3.4. Bayesian Parameter Inference

After the optimal design points are selected and the corresponding noisy observations $D\;=\;{\left\{d^{\left(i\right)},y^{\left(i\right)}\right\}}_{i=1}^{N}$ are collected, we can conduct Bayesian inference for the system parameters, that is, to assess the posterior distribution p(θ|D). The posterior p(θ|D) can be calculated via Bayes’ rule (1). However, as there is no closed-form for the evidence in (1) in many practical problems, the Markov chain Monte Carlo (MCMC) method is often used to generate samples of the posterior distribution. Next, we give a brief review of the MCMC algorithm.

The basic idea of MCMC is to construct a Markov chain over the state space until the chain has reached a stationary distribution, which is assumed to be a target distribution. Here our target distribution is set to be the posterior distribution p(θ|D). Although there are many variants of MCMC, we focus on the Metropolis-Hastings MCMC (MH-MCMC) method [43,44,45]. The basic idea of constructing the Markov chain in MH-MCMC algorithm is that at each step, given current state θ, a candidate state $\theta^{\mathrm{cand}}$ is proposed with probability $q(\theta^{\mathrm{cand}}|\theta )$, where q(·|·) is referred to as the *proposal distribution*. Note that proposal distribution can be arbitrary. A commonly used proposal is the symmetric Gaussian distribution, that is, $q\left(\theta^{\mathrm{cand}}|\theta \right)=N\left(\theta ,\lambda I\right)$, where λ denotes the *stepsize*. Whether to accept the candidate state is determined by the acceptance probability α, given by the following formula,

$$\alpha =\min\left\{1,\frac{p\left(\theta^{\mathrm{cand}}|D\right)/q\left(\theta^{\mathrm{cand}}|\theta \right)}{p\left(\theta |D\right)/q\left(\theta |\theta^{\mathrm{cand}}\right)}=\frac{p(\theta^{\mathrm{cand}}|D)}{p(\theta |D)}\right\}\;.$$

Note that the equation holds only when the proposal distribution is symmetric. If $p\left(\theta^{\mathrm{cand}}|D\right)/p\left(\theta |D\right)>1$, it means that $\theta^{\mathrm{cand}}$ is more possible than the current state θ, we accept the proposal with probability α=1. Otherwise, we accept the proposal with probability α. The detailed MH-MCMC algorithm is summarized in Appendix B.

## 4. Numerical Experiments

In this section, we consider three numerical examples: a standard linear Gaussian model, a nonlinear simple model, and a partial differential equation (PDE) model. Since the first problem has analytical expressions, we examine the performance of our method by comparing the numerical result with the exact solution. The second problem is a commonly-used test problem, and we demonstrate the efficiency of our method through it. In the third test problem, a physical system governed by the diffusion equation is considered, in which a contaminant source inversion problem is studied.

### 4.1. Test Problem 1: Linear Gaussian Problem

We consider the standard linear Gaussian problem in the following form,

$$G\left(\theta ,d\right)=\theta^{T}d\;,\;y=G\left(\theta ,d\right)+\epsilon \;,$$

where the noise and the prior are assumed to be Gaussian, $\epsilon ∼N(0,\sigma^{2})\;,$$\theta ∼N(0,I_{n_{\theta }\times n_{\theta }})$ and $n_{\theta }=n_{d}$. The posterior distribution is a multivariate Gaussian distribution

$$\begin{array}{c} \theta |d,y∼N(\bar{\theta },\bar{\Sigma }), \end{array}$$

where the mean and the covariance are

$$\begin{array}{c} \bar{\theta }=\frac{y}{\sigma^{2}}\bar{\Sigma }d\;,\;\bar{\Sigma }={\left(\frac{dd^{T}}{\sigma^{2}}+I\right)}^{-1}. \end{array}$$

The expected information gain (EIG) for θ can then be given in a closed form. (The detailed deduction is shown in Appendix A.),

(13) $$U\left(d\right)=-\frac{1}{2}\log\left[\det\left(\bar{\Sigma }\right)\right]\;.$$

Note that maximizing EIG is equivalent to minimizing the determinant of the posterior covariance matrix [11] (the Bayesian D-optimal design).

**Figure 1:**
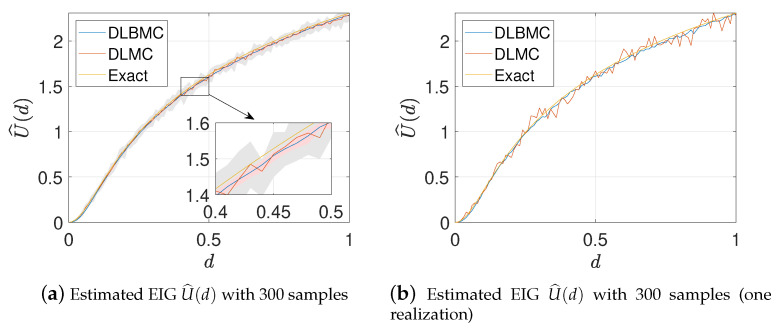
Estimated expected information gain (EIG) profile over the design space for test problem 1. (**a**) Pink and gray shaded areas represent the interval containing 80% of 20 independent estimates of two estimators at each *d* respectively. Blue line and red line indicates the means of estimates. (**b**) One set of realizations of the two estimators.

**Figure 2:**
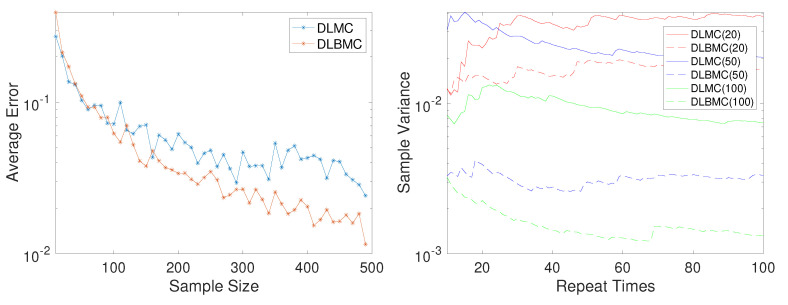
(**Left**) Error averaging over n=20 trails versus the sample size of DLMC and DLBMC. (**Right**) Sample variance versus the repeat times for different sample sizes of double-loop Monte Carlo (DLMC) and double-loop Bayesian Monte Carlo (DLBMC) (numbers in the parenthesis indicate the sample sizes).

We consider the one-dimensional case where $n_{\theta }=1$, d∈[0,1], and set the standard deviation of the noise to σ=0.1. The hyperparameters of DLBMC are set to $l_{\mathrm{in}}=0.5,{\left(\sigma_{f}\right)}_{\mathrm{in}}=1,$ and $l_{\mathrm{out}}=0.2,{\left(\sigma_{f}\right)}_{\mathrm{out}}=1.$ Figure 1 shows the exact EIG, the estimated EIG using DLMC with 300 samples, and the estimated EIG using DLBMC with 300 samples. It can be seen that the estimated EIG of DLBMC is more accurate and more stable than that of DLMC. Besides, for d=0.3, the relationship between the sample size and the bias of the DLMC and DLBMC estimators is studied. As the sample size increases, we compute the bias of the DLBMC estimator and the DLMC estimator averaging 20 trails respectively. Figure 2(Left) shows the results of the bias, where it can be seen that the DLBMC estimator converges to the true value faster than the DLMC estimator, and DLBMC is more accurate than DLMC.

As discussed in Section 3.2, the variance of the BMC estimator for the uniform distribution is not explicitly given. We use the sample variance as an alternative to compare the discrepancy of the DLMC estimator and the DLBMC estimator. Fixing the design point d=0.5, for different sample sizes of DLMC and DLBMC, we repeatedly compute the estimator $\hat{U}\left(d\right)$ *n* times, and denote them by ${\left\{{\hat{U}}^{\left(i\right)}\right\}}_{i=1}^{n}$. Let $\bar{U}\left(d\right)$ denote the mean estimator, $\bar{U}\left(d\right):=\frac{1}{n}\sum_{i=1}^{n}{\hat{U}}^{\left(i\right)}\left(d\right)$, and then the sample variance estimator is defined as

$$s^{2}:=\frac{\sum_{i=1}^{n}\left({\hat{U}}^{\left(i\right)}\left(d\right)-\bar{U}\left(d\right)\right)}{n-1}\;.$$

As the number of repeat times *n* increases, we compare the sample variance of two estimators with different sample sizes for DLMC and DLBMC in Figure 2(Right). It is clear that the DLBMC estimator outperforms the DLMC estimator.

### 4.2. Test Problem 2: Nonlinear Simple Problem

In this section, a simple nonlinear model is tested, which is also studied in [14]. This model is written as

$$G\left(\theta ,d\right)=\theta^{3}d^{2}+\theta \exp\left(-|0.2-d|\right)\;,$$

the prior is set to θ∼U(0,1), and the standard deviation of the observation noise is set to σ=0.01. The hyperparameters of DLBMC are set to $l_{\mathrm{in}}=0.01,{\left(\sigma_{f}\right)}_{\mathrm{in}}=0.2,$ and $l_{\mathrm{out}}=0.05,{\left(\sigma_{f}\right)}_{\mathrm{out}}=0.2.$

**Figure 3:**
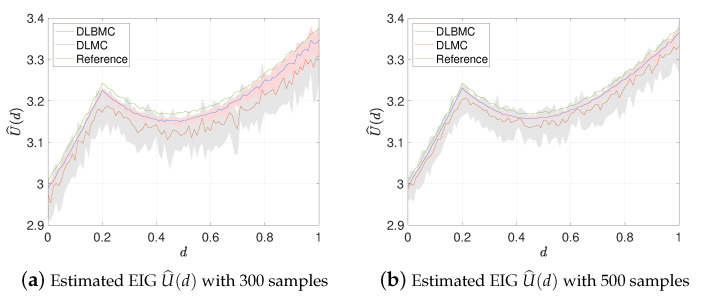
Estimated EIG profile over the design space for test problem 2. Pink and gray shaded areas represent the interval containing 80% of 20 independent estimates of EIG at each *d* for DLBMC and DLMC respectively. Blue line and red line denotes the means of these estimators. Green line denotes the reference solution given by DLMC with $10^{5}$ samples.

Figure 3a,b show the estimated EIG using DLMC and DLBMC with 300 and 500 samples respectively. A reference solution using DLMC with $10^{5}$ samples is also compared in Figure 3a,b. Here, 20 trails of the DLMC estimator and the DLBMC estimator are generated, and Figure 3 shows the mean estimates and the intervals containing 80% of the trails. It is clear that, compared to DLMC, our DLBMC estimator has smaller variances. Compared to the reference solution, DLBMC gives biased estimation. With the increasing sample size, the extent of bias is reduced as we expect.

**Figure 4:**
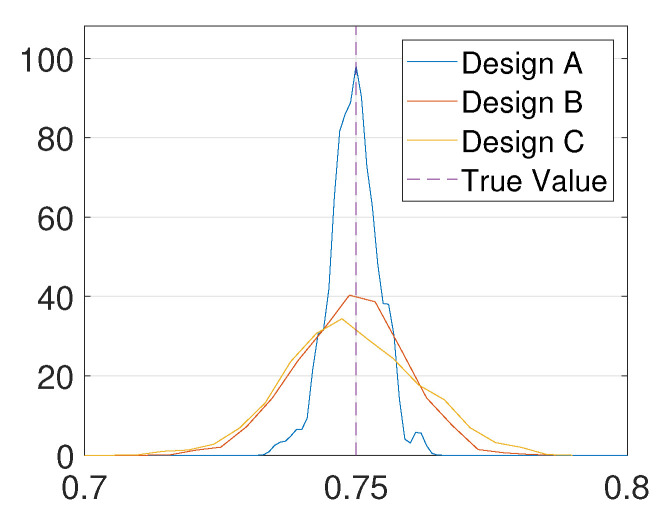
Posterior density functions given by different designs.

To illustrate the effect of optimal design, we compare the posterior distribution given by three design points. Let design A = 1 be the optimal design point, let design B = 0.2 be the local maximizer of the EIG, and let design C = 0 which has the least information since it has the least EIG value. The MH-MCMC algorithm (Algorithm A1) with $N_{\mathrm{iter}}=1000$ and γ=0.2 is used to generate samples of the posterior distribution, and kernel density estimation is used to obtain the posterior density functions from these samples. For this test problem, the ground-truth is set to 0.75. From Figure 4, it can be seen that the posterior distribution obtained through design A is the most accurate, and it has the smallest variance.

### 4.3. Test Problem 3: Source Inversion for the Diffusion Problem

Letting $D\subset R^{2}$ be a bounded and connected domain with a polygonal boundary ∂D, the governing equation of the diffusion problem studied in this test problem is: find a random function u(x,ω)∈D×Ω→R, such that P-a.e. in Ω,

(14) $$\begin{array}{cc} -\nabla^{2}u\left(x,\omega \right)=f\left(x,\omega \right)\;, & \mathrm{in}\;D\;, \end{array}$$

(15) $$\begin{array}{cc} u(x,\omega )=0\;, & \mathrm{on}\;\partial D\;, \end{array}$$

where (Ω,F,P) is a probability space. We consider a square physical domain $D=\left[-1,1\right]\times \left[-1,1\right]\subset R^{2}$, and u(x,ω) in (14)–(15) denotes the concentration of a contaminant at the point x∈D. Let f(x,ω) denote the field of contaminant source. As *f* is usually strictly positive following the setting in [13,46,47,48], we set the prior distribution of f(x,ω) to a log-normal random field, that is, f(x,ω)=exp(a(x,ω)) where a(x,ω) is a normal random field. In this study, the experimental goal is to infer the underlying contaminant field *f* given several observations ${\left\{x_{i},y_{i}\right\}}_{i=1}^{K}$, where design variable $x_{i}$ denotes *i*-th sensor placement, the response is the corresponding numerical PDE solution $u(x_{i})$ with additional noise, that is, $y_{i}=u\left(x_{i}\right)+\epsilon_{i}$, $\epsilon_{i}∼N\left(0,\sigma^{2}\right)$, and *K* is the number of sensors. In this test problem, the hyperparameters of DLBMC are set to $l_{\mathrm{in}}=0.02,{\left(\sigma_{f}\right)}_{\mathrm{in}}=0.01,$ and $l_{\mathrm{out}}=0.005,{\left(\sigma_{f}\right)}_{\mathrm{out}}=0.005.$

We parameterize the permeability field log[f(x,ω)] by a truncated Karhunen-Loève (KL) expansion. Consider the random field a(x,ω)=log[f(x,ω)] with mean function $a_{0}\left(x\right)$, standard deviation σ and covariance function C(x,y),

(16) $$C\left(x,y\right)=\sigma \exp\left(-\frac{|x_{1}-y_{1}|}{c}-\frac{|x_{2}-y_{2}|}{c}\right)\;,$$

where *c* is the correlation length. Then the truncated KL expansion of *f* is expressed as

(17) $$f\left(x,\omega \right)\approx \exp\left(a_{0}\left(x\right)+\sum_{n=0}^{M}\sqrt{\lambda_{n}}\xi_{n}a_{n}\left(x\right)\right)\;,$$

where $a_{n}\left(x\right)$ and $\lambda_{n}$ are the eigenfunctions and eigenvalues of (16) and ${\left\{\xi_{n}\right\}}_{n=1}^{M}$ are uncorrelated random variables. The prior of ${\left\{\xi_{n}\right\}}_{n=1}^{M}$ are set to be independent standard normal distributions, $\xi_{n}∼N\left(0,1\right)$ for n=1,…,M.

In the numerical experiment, we set $a_{0}\left(x\right)=1$, c=2 and σ=1. Fixing the hyperparameters in the truncated KL expansion, the response depends on the random variables ${\left\{\xi_{i}\right\}}_{i=1}^{M}$. Therefore we define the parameter of interest as $\theta :=[\xi_{1},\ldots ,\xi_{M}]$, and rewrite the governing diffusion equation as,

(18) $$\begin{array}{cc} -\nabla^{2}u\left(x,\theta \right)=f\left(x,\theta \right)\;, & \mathrm{in}\;D\times \Gamma \;, \end{array}$$

(19) $$\begin{array}{cc} u(x,\theta )=0\;, & \mathrm{on}\;\partial D\times \Gamma \;. \end{array}$$

We use the bilinear finite element method (FEM) to discretize the diffusion equation over a 17×17 square grid and let the standard deviation of noise be 1% of the mean observed value. Supposing *K* sensors are placed over the design space, generally, we can perform a batch design, and write the following altered forward model

$$\left[\begin{array}{c} y_{1}\\ \vdots \\ y_{i}\\ \vdots \\ y_{K} \end{array}\right]=\left[\begin{array}{c} G(d_{1},\theta )\\ \vdots \\ G(d_{i},\theta )\\ \vdots \\ G(d_{K},\theta ) \end{array}\right]+\left[\begin{array}{c} \epsilon_{1}\\ \vdots \\ \epsilon_{i}\\ \vdots \\ \epsilon_{K} \end{array}\right]=G\left(d_{1:K},\theta \right)+\epsilon \;,$$

where the subscript *i* denotes the *i*-th design variable for i=1,…,K. Directly maximing over the EIG with altered forward model can give optimal design in the context of batch design.

Let $f_{\mathrm{truth}}$ denote the underlying true permeability field. Suppose we have collected data on *K* sensors, and then we perform MCMC to get samples ${\left\{\theta^{\left(i\right)}\right\}}_{i=1}^{N_{\mathrm{iter}}}$ of the posterior distribution using Algorithm A1. In this test problem, we set $N_{\mathrm{iter}}=4000$. Two useful statistics can be obtained from the samples—the maximum a posterior (MAP) estimate $\theta_{\mathrm{MAP}}$ and the mean estimate $\theta_{\mathrm{MEAN}}$. Let $f_{\mathrm{MAP}}$ and $f_{\mathrm{MEAN}}$ be the source fields generated by $\theta_{\mathrm{MAP}}$ and $\theta_{\mathrm{MEAN}}$ respectively. To test the accuracy of the inversion, we introduce the following relative errors

$$\begin{array}{cc} \; & E_{\mathrm{MAP}}=\frac{∥f_{\mathrm{MAP}}-f_{\mathrm{truth}}{∥}_{2}}{∥f_{\mathrm{truth}}{∥}_{2}}\;,\\ \; & E_{\mathrm{MEAN}}=\frac{∥f_{\mathrm{MEAN}}-f_{\mathrm{truth}}{∥}_{2}}{∥f_{\mathrm{truth}}{∥}_{2}}\;, \end{array}$$

where $f_{\mathrm{truth}},f_{\mathrm{MAP}}$ and $f_{\mathrm{MEAN}}$ are discretized over the FEM grids for computational simplicity and ${∥\cdot ∥}_{2}$ denotes the $l^{2}$ vector norm.

Noting that performing grid search over ${\left(17\times 17\right)}^{K}$ grid is computationally expensive, we utilize Bayesian optimization method to efficiently find the optimal designs within a few iterations. Three cases of *K* are considered in the following, which are K=1,2,3.

**Figure 5:**
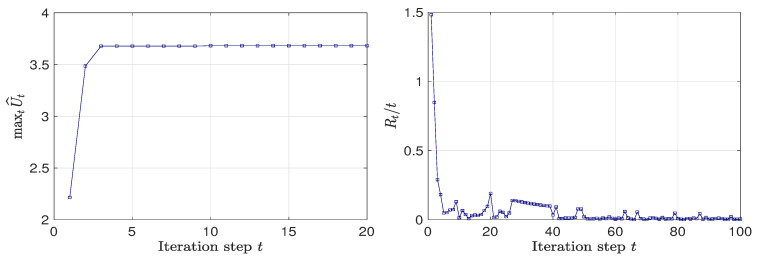
(**Left**) Maximum EIG value at iteration *t* of Bayesian optimization. (**Right**) Average cumulative regret at iteration *t* of Bayesian optimization. K=1, test problem 3.

First, for K=1, we first perform Bayesian optimization over a 17×17 grid. The performance of Bayesian optimization is shown in Figure 5. In Figure 5(Left), we can see that, Bayesian optimization can find the maximum of the EIG within a few iterations. The optimal design found by Bayesian optimization is [0,0], which is expected due to the symmetry of the forward model. In this case, as the number of gird points (289 points) is not too large, we can verify that the optimal design is [0,0] through grid search. Figure 5(Right) shows that the average cumulative regret is convergent to zero.

**Figure 6:**
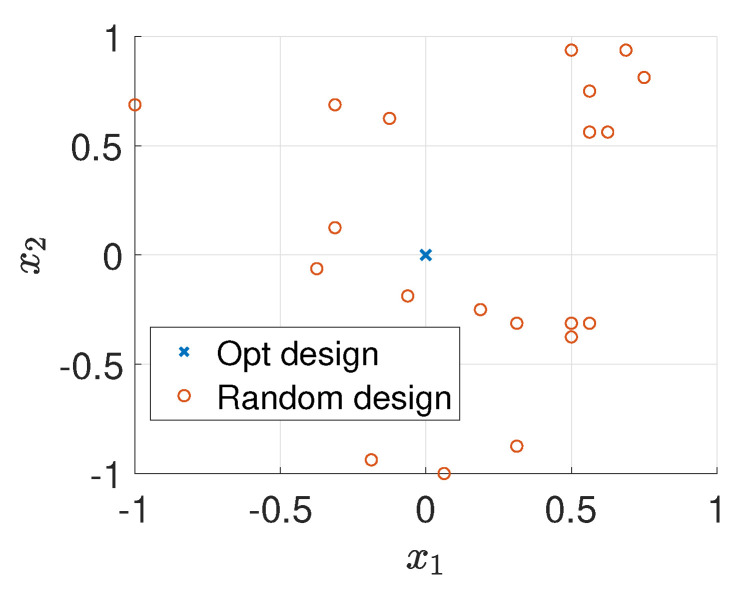
Sensor locations for K=1, test problem 3.

**Figure 7:**
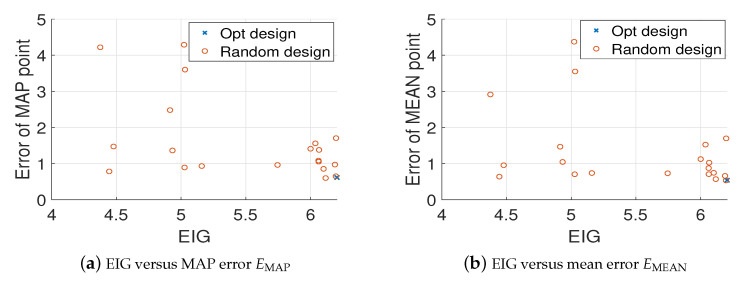
Value of EIG versus the relative errors averaged over 20 trails, K=1, test problem 3.

For comparison, we randomly generate 20 different design points and use the MH-MCMC algorithm to generate 4000 samples of the posterior distribution. Figure 6 shows the locations of the optimal design and the 20 random designs. Figure 7a,b show the relative errors of MAP and mean estimates of the source field (averaged over 20 trails) versus the values of EIG, where it is clear that as the value of EIG becomes larger, the errors of both MAP and mean estimates reduce. In addition, the optimal design point gives large EIG values and relatively small errors. Although the errors associated with the optimal design point are typically smaller than the errors associated with the random design points. They are still large, and the estimated source fields are not accurate enough. Therefore, we next consider more design points.

**Figure 8:**
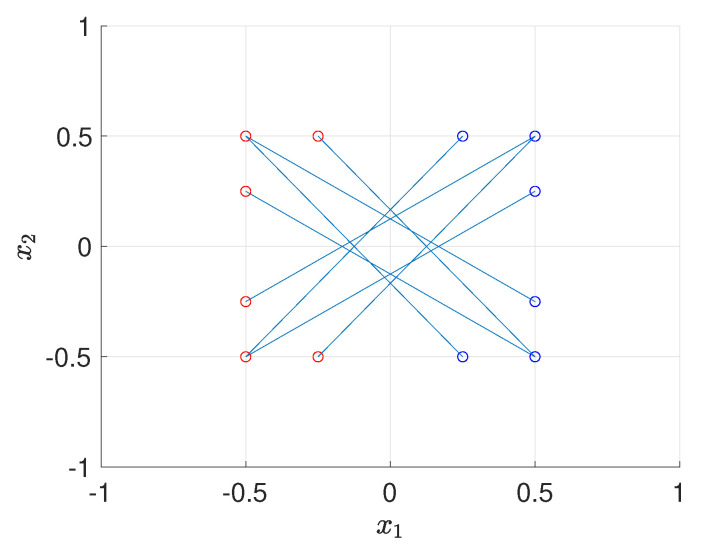
Optimal design (each line connecting blue circle and red circle represents a pair of optimal design points), K=2, test problem 3.

For the multiple design cases (K=2,3), given the fact that the computational cost of batch design increases exponentially as *K* increases, we perform Bayesian optimization over a uniform 9×9 coarse grid. For K≥2, it can be seen that the optimal solution is not unique due to the symmetry of the forward model, for example, $\left[x_{1};x_{2}\right]$ and $\left[-x_{1};-x_{2}\right]$ share the same EIG value. After performing Bayesian optimization several times with BO budget $t_{\max}=100$, the sets of optimal designs for K=2 case are shown in Figure 8, where each line connecting blue circle and red circle represents a pair of optimal design. The numerical result shows that, compared with a pair of two design points that are symmetric with respect to [0;0], a pair of slightly skewed design points can provide more information.

**Figure 9:**
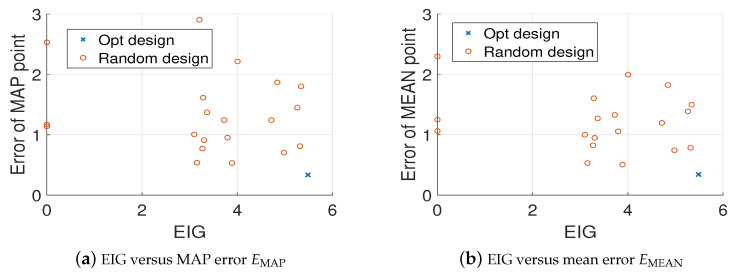
Value of EIG versus the relative errors averaged over 20 trails, K=2, test problem 3.

**Figure 10:**
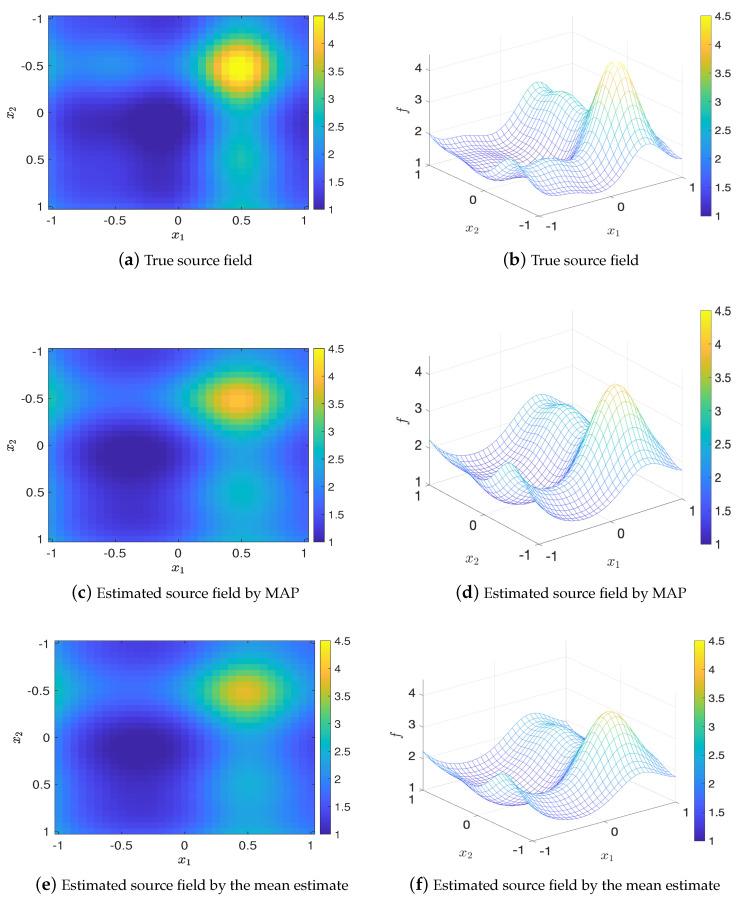
Comparison of the true source field and estimated source fields by MAP and mean estimates for K=2, test problem 3.

Again, we randomly generate 20 sets of design points, and compare them with the optimal design (we choose [0.5,0.5;−0.5,−0.25]). Figure 9 shows the errors of MAP and mean estimates (averaged over 20 trails) of the source field versus the values of EIG, where it is clear that the optimal design leads to the largest EIG value and the smallest error. Besides, the comparison of true source field and the fields generated by MAP and mean estimates are presented in Figure 10. It can be seen that the estimated source field associated with the optimal design matches the true source field well.

**Figure 11:**
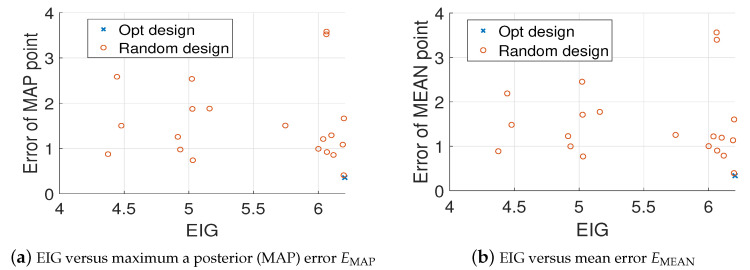
Value of EIG versus the relative errors averaged over 20 trails, K=3, test problem 3.

**Figure 12:**
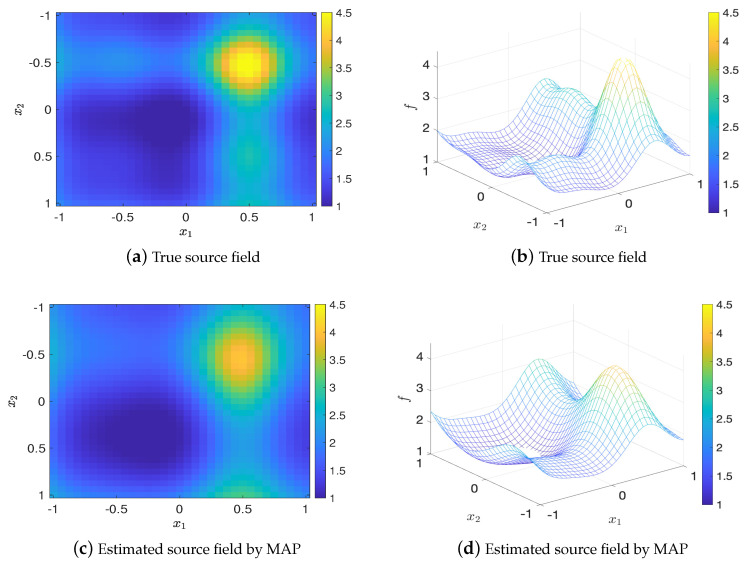

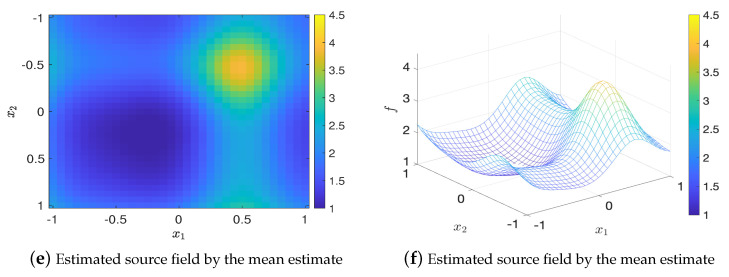
Comparison of the true source field and estimated source fields by MAP and mean estimates for K=3, test problem 3.

For K=3, let the BO budget $t_{\max}=100$, the set of optimal design that found by Algorithm 2 is [0.75,0.25;0.5,−0.25;−0.75,−0.25]. Figure 11 shows the values of EIG and the relative errors in MAP and mean estimates (averaged over 20 trails) for the optimal design and twenty random designs, where it can be seen that the optimal design has the largest EIG value and the smallest errors which are consistent with the results for K=1,2. Figure 12 shows that the estimated source fields generated by the MAP and the mean estimates match the true source field well.

**Figure 13:**
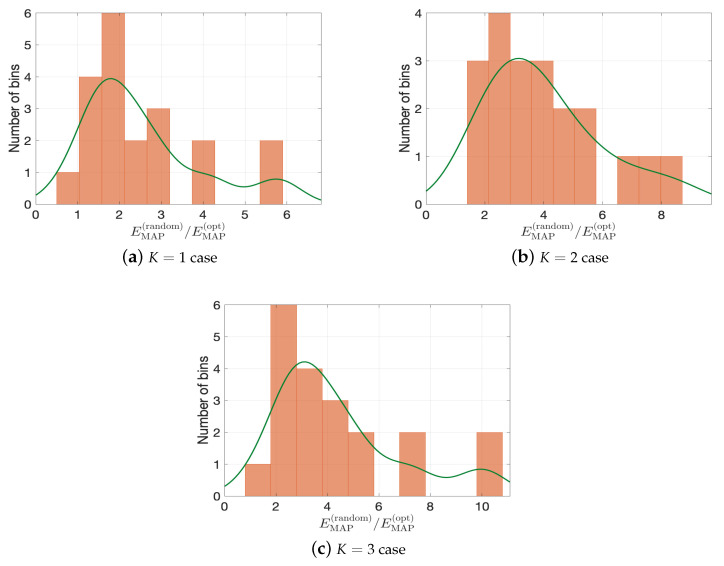
Histograms of $E_{\mathrm{MAP}}^{\left(\mathrm{random}\right)}/E_{\mathrm{MAP}}^{\left(\mathrm{opt}\right)}$. Green lines denotes the kernel smoothing function estimates.

To further quantify the performance of optimal designs, we compute the ratio of $E_{\mathrm{MAP}}$ of random designs and $E_{\mathrm{MAP}}$ of optimal designs (denoted as $E_{\mathrm{MAP}}^{\left(\mathrm{random}\right)}$ and $E_{\mathrm{MAP}}^{\left(\mathrm{opt}\right)}$ respectively) for K=1,2,3 cases. Figure 13 presents the histograms of ratio of relative errors (i.e., $E_{\mathrm{MAP}}^{\left(\mathrm{random}\right)}/E_{\mathrm{MAP}}^{\left(\mathrm{opt}\right)}$), where the green lines are the corresponding kernel smoothing function estimates. We can see that, in all three cases, with high probability, the optimal design can give better posterior performance than the random designs. Especially, from Figure 13c, the error of random designs can be ten times greater than that of the optimal design.

## 5. Conclusions and Discussion

Efficiently using a small number of samples to reduce the cost of computing the expected information gain (EIG) is a fundamental concept to solve the challenging Bayesian optimal experimental design problem. Based on the Bayesian Monte Carlo (BMC) method, a novel double-loop Bayesian Monte Carlo (DLBMC) estimator is proposed for evaluating the EIG in this work. To result in an efficient overall optimization procedure to find the maximizer of the EIG, a Bayesian optimization (BO) procedure for EIG is developed. In addition, our analysis gives explicit expressions of the mean estimate of the BMC estimator and the bounds of its variance for the uniform and the normal distributions. Detailed numerical studies show that our DLBMC method can provide accurate mean estimates with small variances, and the overall BO procedure leads to optimal designs which give efficient Bayesian inference results.

As our novel DLBMC estimator for EIG is based on Gaussian process, it is currently limited to low-dimensional problems where the number of design variables is not large. In this work, the classical BO and MCMC approaches are used, and it is not straight forward to apply them for high-dimensional problems. For high-dimensional Bayesian optimal design problems with a large number of design variables, a possible solution is to conduct a sequential design procedure, and apply DLBMC at each step in the sequential procedure. Conducting a systematic DLBMC based on the sequential design will be the focus of our future work.

## Appendix A. Deduction of Linear Gaussian Model

We consider the standard linear Gaussian model,

$$G\left(\theta ,d\right)=\theta^{T}d\;,\;y=G\left(\theta ,d\right)+\epsilon \;,$$

where the noise and prior are assumed to be Gaussian, $\epsilon ∼N(0,\sigma_{n}^{2})\;,$ and $\theta ∼N(0,I_{n\times n})$.

$$p\left(\theta \right)=\frac{1}{{\left(2\pi \right)}^{n/2}}\exp\left(-\frac{1}{2}\theta^{T}\theta \right)\;.$$

then the likelihood is given by

$$\begin{array}{cc} p(y|d,\theta )\; & =\frac{1}{\sqrt{2\pi }\sigma_{n}}\exp\left(-\frac{{\left(y-G\left(d,\theta \right)\right)}^{2}}{2\sigma_{n}^{2}}\right)\\ \; & =\frac{1}{\sqrt{2\pi }\sigma_{n}}\exp\left(-\frac{{\left(y-\theta^{T}d\right)}^{2}}{2\sigma_{n}^{2}}\right)\;. \end{array}$$

The posterior is given by

$$\begin{array}{cc} \; & p\left(\theta |y,d\right)=\frac{p(y|\theta ,d)p(\theta )}{p(y|d)}\propto p\left(y|\theta ,d\right)p\left(\theta \right)\\ \; & \;\propto \exp\left[-\frac{{\left(y-\theta^{T}d\right)}^{2}}{2\sigma_{n}^{2}}-\frac{\theta^{T}\theta }{2}\right]\\ \; & \;=\exp\left[-\frac{1}{2}\theta^{T}\left(\frac{dd^{T}}{\sigma_{n}^{2}}+I\right)\theta +\frac{y}{\sigma_{n}^{2}}\theta^{T}d+\frac{y^{2}}{2\sigma_{n}^{2}}\right]\\ \; & \;\propto \exp\left[-\frac{1}{2}{\left(\theta -\bar{\theta }\right)}^{T}\left(\frac{dd^{T}}{\sigma_{n}^{2}}+I\right)\left(\theta -\bar{\theta }\right)\right]\;, \end{array}$$

where

$$\bar{\theta }=\frac{y}{\sigma_{n}^{2}}{\left(\frac{dd^{T}}{\sigma_{n}^{2}}+I\right)}^{-1}d\;,\;\bar{\Sigma }={\left(\frac{dd^{T}}{\sigma_{n}^{2}}+I\right)}^{-1}\;.$$

By the definition of expected information gain,

$$\begin{array}{cc} U(d) & =\int_{Y}\int_{\Theta }\log\left[\frac{p(\theta |y,d)}{p(\theta )}\right]p\left(\theta |y,d\right)d\theta dy\;. \end{array}$$

Supposing $\theta |y,d∼N(\bar{\theta },\bar{\Sigma })$, then we have

$$\begin{array}{c} \frac{p(\theta |y,d)}{p(\theta )}=\frac{1}{{\left(\det\bar{\Sigma }\right)}^{1/2}}\exp\left(-\frac{\left(\theta -\bar{\theta }\right)\Sigma^{-1}\left(\theta -\bar{\theta }\right)}{2}+\frac{\theta^{T}\theta }{2}\right)\;. \end{array}$$

Thus,

$$\begin{array}{c} \log\frac{p(\theta |y,d)}{p(\theta )}=-\frac{1}{2}\ln\left(\det\bar{\Sigma }\right)-\frac{\left(\theta -\bar{\theta }\right)\Sigma^{-1}\left(\theta -\bar{\theta }\right)}{2}+\frac{\theta^{T}\theta }{2}. \end{array}$$

Below, we show that

$$\begin{array}{cc} \; & \int_{y}\int_{\Theta }\frac{\left(\theta -\bar{\theta }\right)\Sigma^{-1}\left(\theta -\bar{\theta }\right)}{2}p\left(\theta |y,d\right)d\theta p\left(y|d\right)dy\\ \; & \;=\int_{y}\int_{\Theta }\frac{\theta^{T}\theta }{2}p\left(\theta |y,d\right)d\theta p\left(y|d\right)dy. \end{array}$$

Let $z=\Sigma^{-1/2}\theta$, which can be viewed as a linear transformation of a random vector. We have that $\bar{z}=\Sigma^{-1/2}\bar{\theta }$, and the covariance of *z* is

(A1) $$\begin{array}{cc} V(z) & =E[\left(z-\bar{z}\right){\left(z-\bar{z}\right)}^{T}]\\ \; & =\Sigma^{-1/2}E\left[\left(\theta -\bar{\theta }\right){\left(\theta -\bar{\theta }\right)}^{T}\right]\Sigma^{-1/2}=I. \end{array}$$

Taking (A1) at hand, the following equation can be obtained

$$\begin{array}{cc} \; & \int_{\Theta }\frac{\left(\theta -\bar{\theta }\right)\Sigma^{-1}\left(\theta -\bar{\theta }\right)}{2}p\left(\theta |y,d\right)d\theta =\int_{Z}\frac{{\left(z-\bar{z}\right)}^{T}\left(z-\bar{z}\right)}{2}p\left(z|y,d\right)dz\\ \; & \;=\sum_{i=1}^{n}E\left[{\left(z_{i}-{\bar{z}}_{i}\right)}^{2}\right]/2=n/2. \end{array}$$

Then we also have

$$\begin{array}{c} \int_{y}\int_{\Theta }\frac{\left(\theta -\bar{\theta }\right)\Sigma^{-1}\left(\theta -\bar{\theta }\right)}{2}p\left(\theta |y,d\right)d\theta p\left(y|d\right)dy=n/2. \end{array}$$

Next, we consider the integration $\int_{y}\int_{\Theta }\frac{\theta^{T}\theta }{2}p\left(\theta |y,d\right)d\theta p\left(y|d\right)dy$. Since p(θ|y,d)p(y|d)=p(θ)p(y|θ,d),

$$\begin{array}{cc} \; & \int_{y}\int_{\Theta }\frac{\theta^{T}\theta }{2}p\left(\theta |y,d\right)d\theta p\left(y|d\right)dy=\int_{y}\int_{\Theta }\frac{\theta^{T}\theta }{2}p\left(\theta \right)d\theta p\left(y|\theta ,d\right)dy\\ \; & \;=\int_{\Theta }\frac{\theta^{T}\theta }{2}p\left(\theta \right)\int_{y}p\left(y|\theta ,d\right)dyd\theta =\int_{\Theta }\frac{\theta^{T}\theta }{2}p\left(\theta \right)d\theta =n/2. \end{array}$$

Therefore we have the analytical form of EIG,

(A2) $$U\left(d\right)=-\frac{1}{2}\log\left[\det\left(\bar{\Sigma }\right)\right]\;.$$

## Appendix B. Metropolis-Hastings MCMC Algorithm

**Algorithm A1** The Metropolis-Hastings Markov chain Monte Carlo (MH-MCMC) algorithm
1: **Input:** Prior p(θ), stepsize γ, maximum number of iterations $N_{\mathrm{iter}}$. 2: **Initialize:**$\theta^{\left(0\right)}∼p\left(\theta \right)$. 3: **for** $i=1,2,\ldots ,N_{\mathrm{iter}}$ **do** 4: Propose: $\theta^{\mathrm{cand}}=\theta^{\left(i-1\right)}+\gamma N\left(0,I\right)\;.$ 5: Acceptance Probability: 6: $\;\;\alpha \left(\theta^{\mathrm{cand}}|\theta^{\left(i-1\right)}\right)=\min\left\{1,\frac{p(\theta^{\mathrm{cand}}|D)}{p(\theta^{\left(i-1\right)}|D)}\right\}\;.$ 7: Draw u∼U(0,1). 8: **if** u<α **then** 9: Accept the proposal: $\theta^{\left(i\right)}\leftarrow \theta^{\mathrm{cand}}$. 10: **else** 11: Reject the proposoal: $\theta^{\left(i\right)}\leftarrow \theta^{\left(i-1\right)}$. 12: **end if** 13: **end for**
